# At the Crossroads: Does the Configuration of Roadside Vegetation Affect Woodland Bird Communities in Rural Landscapes?

**DOI:** 10.1371/journal.pone.0155219

**Published:** 2016-05-16

**Authors:** Mark Hall, Dale Nimmo, Andrew F. Bennett

**Affiliations:** 1 School of Life and Environmental Sciences, Deakin University, Burwood, 3125, Victoria, Australia; 2 Department of Ecology, Environment and Evolution, La Trobe University, Bundoora, 3086, Victoria, Australia; 3 Institute for Land, Water and Society, School of Environmental Science, Charles Sturt University, Albury, 2640, New South Wales, Australia; 4 Arthur Rylah Institute for Environmental Research, Department of Environment, Land. Water & Planning, 123 Brown St., Heidelberg, Victoria, 3084, Australia; Centre for Cellular and Molecular Biology, INDIA

## Abstract

In agricultural regions worldwide, linear networks of vegetation such as hedges, fencerows and live fences provide habitat for plant and animal species in heavily modified landscapes. In Australia, networks of remnant native vegetation along roadsides are a distinctive feature of many rural landscapes. Here, we investigated the richness and composition of woodland-dependent bird communities in networks of eucalypt woodland vegetation along roadsides, in an agricultural region in which >80% of native woodland and forest vegetation has been cleared. We stratified sites in a) cross sections and b) linear strips of roadside vegetation, to test the influence on woodland birds of site location and configuration in the linear network (the ‘intersection effect’). We also examined the influence of tree size at the site, the amount of wooded vegetation surrounding the site, and the abundance of an aggressive native species, the noisy miner *Manorina melanocephala*. Birds were surveyed at 26 pairs of sites (cross section or linear strip) on four occasions. A total of 66 species was recorded, including 35 woodland species. The richness of woodland bird species was influenced by site configuration, with more species present at cross sections, particularly those with larger trees (>30 cm diameter). However, the strongest influence on species richness was the relative abundance of the noisy miner. The richness of woodland birds at sites where noisy miners were abundant was ~20% of that where miners were absent. These results recognise the value of networks of roadside vegetation as habitat for woodland birds in depleted agricultural landscapes; but highlight that this value is not realised for much of this vast vegetation network because of the dominance of the noisy miner. Nevertheless, roadside vegetation is particularly important where the configuration of networks create nodes that facilitate movement. Globally, the protection, conservation and restoration of such linear networks has an important influence on the persistence of biota within human-dominated landscapes.

## Introduction

Landscape modification to meet human needs for food, fibre and living space is a major influence on global biodiversity [1]. A common legacy of such modification, particularly in agricultural environments, is the creation of networks of linear vegetation [2]; such as hedgerows in Europe [3,4], fencerows in North America [5,6], live fences in southern and central America [7], and roadside vegetation in Australia [8,9]. In highly modified regions, such linear elements potentially play an important role in biodiversity conservation [10,11]. Hedgerows and arable field margins in European farmland, for example, provide nest and roost sites, food resources and movement pathways for birds [4,12]; while in the Americas, fencerows and live fences provide refuge, foraging resources and movement corridors for diverse assemblages of birds, butterflies, bats and beetles [5,7].

The creation of roads and highways is among the most extensive and pervasive form of landscape change on Earth [13]. Roadside vegetation, the vegetation between the road surface and boundary of the road reserve, varies greatly in width and composition [14], but collectively represents a vast linear network [15]. In many regions in Australia, roadside vegetation is comprised of remnant native vegetation including grasslands, shrublands, woodlands or forest [16–18]. Typically, it occurs as strips from 5–30 m in width (e.g. [19]), although in some regions ‘travelling stock reserves’ may be greater than 500 m in width [20].

**Fig 1:**
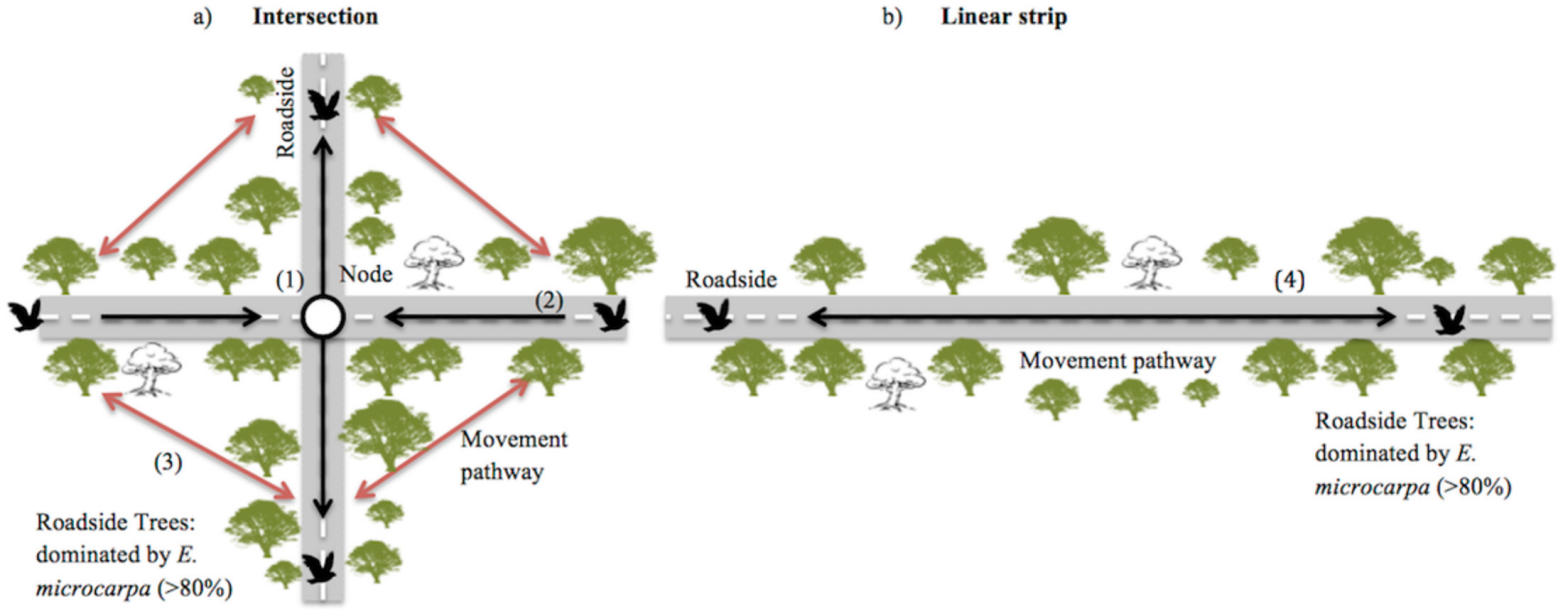
Conceptual model of movement pathways along roadsides in a typical fragmented agricultural landscape: (a) cross section (road intersection), (b) linear strip (straight section of road). (1) The intersection or ‘node’ provides a meeting point of two or more vegetated pathways. It may provide more resources within a smaller, more defendable space for species dependent on wooded vegetation (ie woodland-dependent birds). (2) Cross sections provide multiple movement pathways (in black), enhancing a species ability to reach these nodes of potentially high resources (food, shelter, nest sites) as well as offering multiple movement/escape routes to other areas. (3) The configuration of vegetation surrounding an intersection also influences the likelihood that species will be able to cross spaces diagonally between road sections (in red); more vegetation provides more movement pathways and increases their chances of meeting resource needs. (4) Linear strips provide only two possible (continuously vegetated) movement pathways, potentially limiting a species ability to reach resources and thus potentially making these sites less likely to support species dependent on vegetation networks.

The spatial configuration of linear networks has implications for their value for biota in modified landscapes [21,22]. One aspect of the spatial configuration is the ecological role of intersections, where two or more linear habitats meet within a network. In a study of breeding birds in agricultural environments, Lack [23] found more species at intersections (or ‘nodes’) of hedges compared with straight sections of hedge of the same length. He postulated this was due to intersections making it easier for smaller birds to defend territories, find food, obtain shelter, and have enhanced movement/retreat options. This ‘intersection effect’ (see Fig 1) in hedge networks has been supported by observations of greater species richness of corridor-dependent bird species at or near intersections [24]. Van Langevelde and Grashof-Bokdam [10] modeled bird movement in hedgerow networks and found that species with limited movement ability occurred at higher densities at intersections than in linear strips. They concluded this was likely due to the species’ ability to recolonise intersections more quickly following mortality of other individuals. The intersection effect has also been associated with increased richness of other taxa, including plants [25] and arthropods [26]. Few studies, however, have tested whether there may be a similar intersection effect on faunal occurrence in other types of linear networks that occur worldwide (but see [27]).

Here, we examine the use of roadside vegetation by woodland birds in southeastern Australia, to test whether intersections in networks of remnant roadside vegetation support a greater number of bird species than linear strips. Woodland birds in southern Australia have experienced serious decline and their conservation is of great concern [28,29]. In many regions, forest and woodland vegetation has been extensively cleared (e.g. >80% loss), such that networks of roadside vegetation form a substantial component of the remaining wooded habitat [19].

In addition to examining the hypothesis that roadside configuration affects woodland bird communities, the (1) ‘configuration’ hypothesis, we also examine three alternative hypotheses of drivers of woodland bird communities in roadside networks in the study region. These are (2) the ‘tree size’ hypothesis, which predicts that larger, older trees at a site are important for woodland birds [30], (3) the ‘habitat amount’ hypothesis, which predicts that sites surrounded by a greater amount of tree cover will contain more woodland bird species (based on [31]); and (4) the ‘biotic interaction’ hypothesis, which relates to the influence of an avian competitor, the noisy miner (*Manorina melanocephala)*, known for its negative effects on woodland bird communities in south-eastern Australia [32,33]. Noisy miners aggressively out-compete or exclude smaller insectivorous species and have become abundant, and dominate bird communities, in many fragmented environments. We predicted that sites with more noisy miners will have fewer woodland bird species.

## Methods

This research was undertaken with the approval of Deakin University Animal Ethics permit B8-2012.

### Study area

The study area spans a region of ~10,000 km^2^ of the Victorian Riverina plains in north-central Victoria, Australia. Mean annual rainfall ranges from 500–750 mm, with most rain in winter and spring (Bureau of Meteorology, 2012. http://www.bom.gov.au/climate/data/). The native vegetation of the region is eucalypt woodland dominated by grey box (*Eucalyptus microcarpa*) and yellow box (*E*. *melliodora*) across drier areas of the plains, with river red gum (*E*. *camaldulensis*) common along streams. Canopy height of these major tree species is typically 10–25 m. Vegetation in the region has been extensively cleared or modified, primarily for agriculture: less than 20% of the original tree-cover remains [34], mainly concentrated in a few large forest blocks, but also as extensive linear networks along roads and streams, and as scattered paddock trees [19]. Land use in the region is comprised largely of grazing by domestic stock (sheep, cattle) and mixed cropping (predominantly wheat, canola).

### Site selection

**Fig 2:**
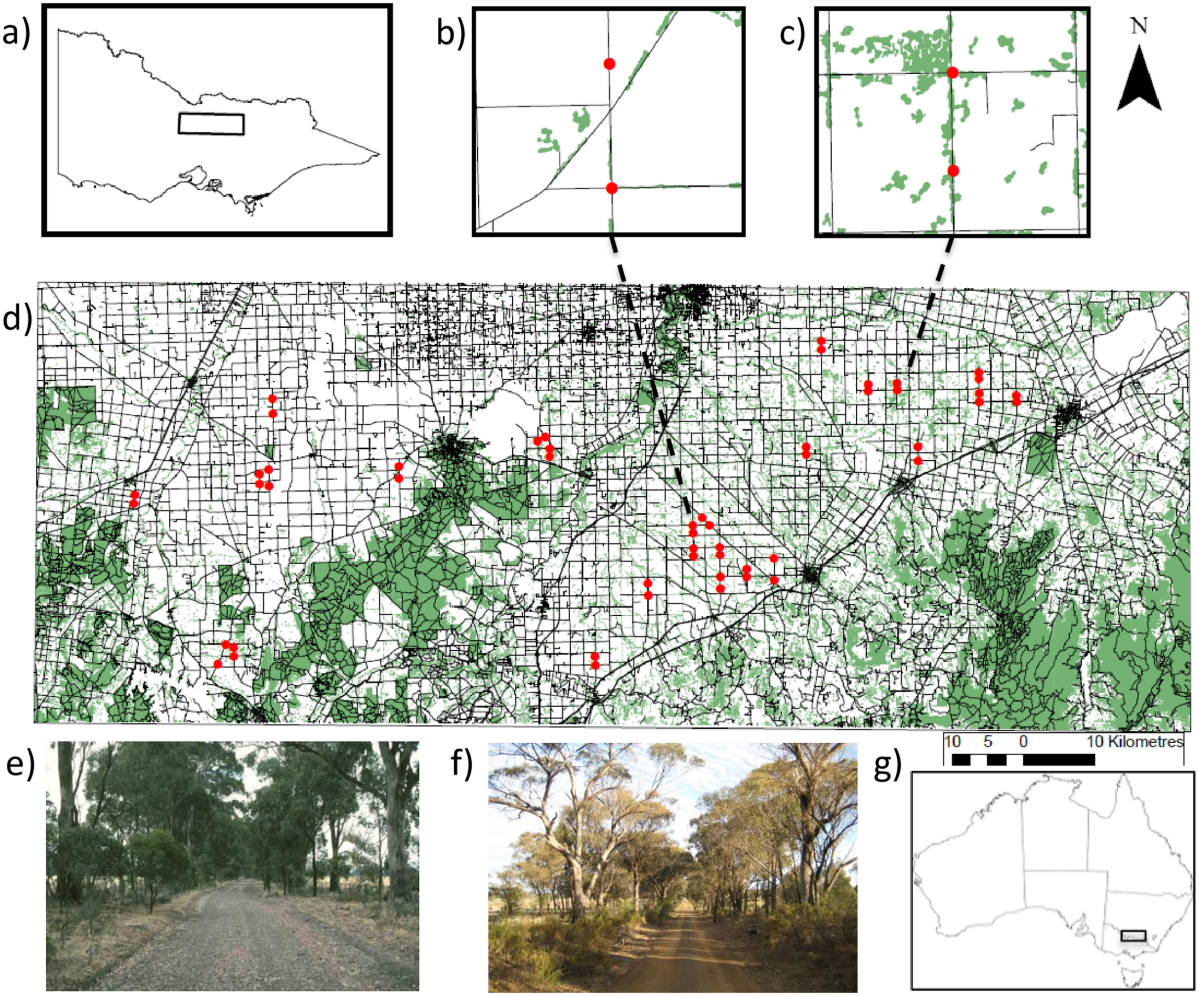
Location of survey sites in north-central Victoria, Australia. Insets (top) show (a) location of study area within Victoria; (b, c) examples of two paired sites (cross section and linear strip) with different levels of surrounding vegetation, and (d) the 52 study sites across the region. Insets (bottom) show examples of two sites, a cross section (e) and linear strip (f), dominated by *E*. *microcarpa*. The study area lies within south-eastern Australia (g).

We used satellite images to identify potentially suitable pairs of sites, each comprising a four-way road intersection (cross section) and an adjacent straight section of road (linear strip). These potential sites were then field-checked to assess whether they met the following requirements. First, sites were set within a roadside vegetation network with canopy gaps of no more than 50 m, and were surrounded by largely cleared farmland. Second, scattered trees and remnant wooded vegetation surrounding sites were visually identified (from satellite images) to represent a range in cover from ~5–30%, typical of this physiographic region. Third, sites were each 1.0 ha in area, and each pair of sites (linear, cross section) was situated along the same road but separated by at least 500 m to enhance independence of samples, and to avoid overlap of surrounding vegetation buffers. Linear sites followed a north-south orientation in relation to the adjoining intersection site. Fourth, to limit the influence of vegetation type and microclimatic conditions on woodland bird communities, suitable sites were chosen to be dominated by a single canopy species, *Eucalyptus microcarpa* (at least 80% by abundance), and were similar in topographic position. If pairs of sites met the above criteria they were retained: 26 pairs (52 sites in total) were selected (Fig 2) from all potentially suitable sites (~80).

Typically, survey transects were 500 m in length and encompassed the 20 m width of the road reserve (i.e. 500 m x 20 m = 1.0 ha), on minor roads originally surveyed as ‘one chain’ roads (22 yards = ~20 m width) [9]. In four instances, roads were ‘two chains’ (~40 m) wide, so the transect was 250 m in length. Secondary and minor roads were chosen to reduce the potential effects of traffic.

### Bird surveys

Bird surveys were conducted at each site for 20 mins (n = 52 sites) in suitable weather conditions (i.e. fine weather, little or no wind). All individuals detected visually or aurally whilst walking the transect midline (along the road) were recorded, and distinction was made between those ‘on site’ and ‘off site’. At intersection sites, transects were walked from south to north for 250 m (10 mins), with the intersection falling at the 125 m mark. The diagonal gap between the north and westerly points was then crossed where possible, to avoid unnecessary disturbance to species by retracing steps. The west-east line was then followed for 250 m (further 10 mins), again with the intersection at the midway point. Care was taken not to record individuals twice near the mid-point.

Surveys were conducted four times at each site on separate days between April and June 2012, by the same observer (MH). Sites were surveyed once each in the early morning, mid-morning, mid-afternoon and late afternoon time periods, respectively, rotated across the study timeframe in a logistically feasible fashion to avoid travel and time delays.

To ensure data reflected potential habitat use at a site, birds flying more than a few metres above the canopy were regarded as off-site; except for aerial foragers such as raptors and swallows, which were included as on-site if they were foraging overhead, and birds flying above the canopy that landed on site. For birds observed flying, the direction in relation to the survey transect (along, across, circling) was recorded to determine patterns in the use of roadside vegetation as potential corridors for movement.

### Vegetation assessment

The species and diameter at breast height (DBH) were recorded for all trees within a randomly selected 0.5 ha section of each transect. The number of juvenile *Eucalypts* was also counted.

### Response and predictor variables

We first grouped bird species to reflect their habitat associations (woodland-dependent, open-country, open-tolerant) [35]. For analyses, we focused on woodland-dependent species because of their dependence on native vegetation. Three response variables were calculated, to represent the number of woodland species at each site (on-site only) in the following categories.

1. Woodland-dependent: the total number of species categorised as woodland-dependent.
2. Resident: the number of woodland species recorded at a site on >50% of surveys.
3. Species’ movement: the proportion of woodland species seen *flying along* the transect in either direction (i.e. parallel with the roadside vegetation, rather than flying across between farmland and roadside vegetation or circling above).

Predictor variables (Table 1) were derived from vegetation data collected at each site, from GIS analysis, and the abundance of noisy miners from surveys at each site. They correspond with the four hypotheses.

**Table 1:** Predictor variables included in models of the richness of *Woodland-dependent* and *Resident* species in roadside vegetation and species’ movements *Flying along* transects.

Hypothesis	Predictor variables	Description
Configuration	Configuration	Sites selected as either cross section (CS) or linear strips (LS)
Tree size	Trees 0–30 cm diameter	Density of small trees (0–30 cm diameter) within the survey transect (stems per ha)
	Trees >30 cm diameter	Density of larger trees (>30 cm diameter) within the survey transect (stems per ha)
Habitat amount	Surrounding vegetation	Amount of wooded vegetation cover within a 500 m radius of the mid-point of each site (ha)
Biotic interaction	Noisy miner	The mean abundance of noisy miners ‘on transect’ over four surveys at each site (individuals per ha)

First, the configuration hypothesis was represented by the main treatment in the study design; that is, a categorical variable with two levels, cross section or linear strip. Second, the tree size hypothesis was represented by counting the density of trees within the survey transect (number per ha) in size categories: 10–30 cm and >30 cm diameter. Saplings smaller than 10 cm diameter were not included. Third, the habitat amount hypothesis was represented by calculating the area of tree cover within buffers of radius 100, 250, 500 and 750 m, surrounding the mid-point of each site. Tree cover was calculated using the Tree25 layer (Department of Environment, Land, Water & Planning, Victoria) in ArcGIS10 (ESRI, 2011). A 500 m buffer was selected because it provided an ecologically meaningful area of surrounding vegetation, did not overlap with buffers of adjoining sites, and provided the strongest fit with the data. Last, the biotic interaction hypothesis was represented by calculating the average abundance (individuals ha^-1^) of the noisy miner from the four surveys at each site.

All predictor variables were standardised to allow a direct comparison of coefficients (mean = 0, standard deviation = 1). Pairwise correlations (Spearman’s rank correlation) between predictor variables were all < 0.55.

### Statistical analysis

For two of the three response variables (woodland-dependent, resident species), generalised linear mixed models (GLMMs) were developed, assuming a Poisson distribution and log-link function. For the species movement (flying along) response variable, GLMMs were developed assuming a binomial distribution and logit-link function. Overdispersion was assessed in the global model. Where the dispersion parameter was >1, an observation-level random effect was fitted to account for additional variance [36]. All models were fitted in R (R_Core_Team, 2012). As sites were spatially paired into treatments (i.e. cross section and linear strip), the pair to which a site belonged was entered as a random effect to account for potential lack of independence. All other environmental variables were regarded as fixed effects [37].

An information theoretic approach was used to compare competing models that represented the four hypotheses and to evaluate the relative support for each model [38]. Candidate models were developed to compare all subsets of hypotheses (i.e. each competing hypothesis and all combinations of hypotheses) (Table 2). We calculated Akaike’s Information Criterion corrected for small sample sizes (AICc) to compare and rank the multiple competing models, and to determine the most parsimonious model [39]. Ranking was undertaken by comparing the AICc difference (Δ_i_) between each model and that with the lowest AICc value (i.e. the ‘best’ model). Models with Δ_i_ ≤2 are considered to have substantial support [38], and those with Δ_i_ of 2–7 have some support and should not necessarily be dismissed [40]. Akaike weights (*w*_i_) were generated to assess the probability that the model is the best of the candidate set [38]. We summed *w*_i_ for the top models to generate a 95% confidence set of the most parsimonious (best fitting) models.

**Table 2:** Results from model averaging for each response variable, showing models within the 95% confidence set. Also shown for each model are the number of parameters (K), AIC_c_ values, AIC_c_ differences (Δ_i_) and Akaike weights (*w*_i_). Variables are described in Table 1.

Group	Model	K	AIC_c_	Δ_i_	*w*_i_	*R*^*2*^
**Woodland dependent**	Configuration + Tree size + Biotic interaction	6	70.88	0.00	0.45	0.53
	Configuration + Tree size + Habitat amount + Biotic interaction	7	72.56	1.68	0.19	0.52
	Configuration + Biotic interaction	4	73.27	2.39	0.14	0.54
**Resident**	Configuration + Biotic interaction	4	55.92	0.00	0.40	0.36
	Biotic interaction	3	57.60	1.69	0.17	0.33
	Configuration + Tree size + Biotic interaction	6	57.90	1.98	0.15	0.38
	Configuration + Habitat amount + Biotic interaction	5	58.00	2.08	0.14	0.36
**Species movement (Flying along)**	Configuration + Habitat amount + Biotic interaction	6	179.30	0.00	0.66	0.49
	Configuration + Biotic Interaction	5	182.83	3.54	0.11	0.45

Summing *w*_i_ for all models within which a particular hypothesis (variable) occurs (∑*w*_i_) gives an importance value ranging from 0–1, indicating the relative importance of that hypothesis in explaining the data [38]. The larger the value, the more importance that hypothesis has relative to others [38]. We summed *w*_i_ for models that included each of the four hypotheses (Table 2) to calculate the probability that each respective hypothesis was in the best model (i.e. the summed Akaike weight, ∑*w*_i_). When no single model was considered ‘clearly best’ (i.e. no models had *w*_i_ >0.90), model averaging was performed using the MuMIn package [41]. We regarded predictor variables as influential when the 95% confidence interval of the model-averaged coefficient did not overlap with zero.

A Morans I test was performed to test for spatial autocorrelation in model residuals for each of the response variables using the spdev package (R_Core_Team, 2015). No spatial autocorrelation was detected (Woodland-dependent species: p = 0.78, Resident species: p = 0.39, Flying along: p = 0.44).

## Results

### Bird species recorded

A total of 66 species was recorded during the four survey rounds (n = 208 surveys in total), including 35 woodland-dependent species (S1 Table, S1 Data). At linear strips (n = 26 sites) 52 species were recorded (27 woodland species), whilst at cross sections (n = 26 sites) 54 species were recorded (28 woodland species) (Table 3).

**Table 3:** Measures of the occurrence of woodland-dependent bird species in relation to site configuration: cross sections (CS) and linear strips (LS) and totals for all sites.

		Cross section			Linear strip			All Sites
*Common name*	*Scientific name*	*# sites (n-26)*	*# surveys (n = 104)*	*Obs*	*# sites (n-26)*	*# surveys (n = 104)*	*Obs*	*Summed obs*
Black-chinned honeyeater	*Melithreptus gularis*	0	0	0	1	1	3	3
Blue-faced honeyeater	*Entomyzon cyanotis*	1	1	1	0	0	0	1
Brown quail	*Coturnix australis*	1	2	5	0	0	0	5
Brown treecreeper	*Climacteris picumnus*	1	4	13	1	4	8	21
Brown-headed honeyeater	*Melithreptus brevirostris*	0	0	0	4	4	48	48
Common bronzewing	*Phaps chalcoptera*	3	5	18	0	0	0	18
Crested shrike-tit	*Falcunculus frontatus*	2	3	5	1	1	2	7
Crimson rosella	*Platycercus elegans*	1	1	1	1	1	2	3
Dusky woodswallow	*Artamus cyanopterus*	1	1	8	0	0	0	8
Eastern spinebill	*Acanthorhynchus tenuirostris*	0	0	0	1	1	1	1
Golden whistler	*Pachycephala pectoralis*	6	11	14	4	4	4	18
Grey fantail	*Rhipidura fuliginosa*	9	16	20	9	13	14	34
Grey shrike-thrush	*Colluricincla harmonica*	6	9	10	6	9	10	20
Grey-crowned babbler	*Pomatostomus temporalis*	4	8	34	3	5	22	56
Jacky winter	*Microeca fascinans*	1	2	5	1	1	1	6
Mistletoebird	*Dicaeum hirundinaceum*	1	1	2	0	0	0	2
Musk lorikeet	*Glossopsitta concinna*	18	41	449	13	24	279	728
Noisy friarbird	*Philemon corniculatus*	1	1	8	0	0	0	8
Olive-backed oriole	*Oriolus sagittatus*	0	0	0	1	1	1	1
Pied currawong	*Strepera graculina*	5	6	8	2	2	3	11
Red wattlebird	*Anthochaera carunculata*	11	20	72	1	16	53	125
Rufous whistler	*Pachycephala rufiventris*	2	2	2	3	4	5	7
Scarlet robin	*Petroica multicolor*	2	4	4	2	2	3	7
Speckled warbler	*Chthonicola sagittata*	1	1	1	0	0	0	1
Spotted pardalote	*Pardalotus punctatus*	1	1	2	1	1	1	3
Superb fairy-wren	*Malurus cyaneus*	3	10	37	2	4	12	49
Tree martin	*Hirundo nigricans*	5	5	24	2	2	18	42
Varied sittella	*Daphoenositta chrysoptera*	3	3	29	3	3	12	41
White-bellied cuckoo-shrike	*Coracina papuensis*	0	0	0	1	1	1	1
White-browed babbler	*Pomatostomus superciliosus*	1	1	5	0	0	0	5
White-plumed honeyeater	*Lichenostomus penicillatus*	15	34	151	16	36	146	297
White-winged chough	*Corcorax melanorhamphos*	13	16	135	6	10	147	282
Yellow thornbill	*Acanthiza nana*	5	9	36	7	14	84	120
Yellow-faced honeyeater	*Lichenostomus chrysops*	0	0	0	1	1	1	1
Yellow-plumed honeyeater	*Lichenostomus ornatus*	0	0	0	1	1	1	1

The most common species included the eastern rosella (*Platycercus eximius*), Australian magpie (*Gymnorhina tibicen*) and galah (*Eolophus roseicapillus*), all suited to open country landscapes with sparse tree cover (S1 Table). Some of the least common species were woodland birds, such as the black-chinned honeyeater (*Melithreptus gularis*), eastern spinebill (*Acanthorhynchus tenuirostris*), crested shrike-tit (*Falcunculus frontatus*) and rufous whistler (*Pachycephala rufiventris*). Of the 35 woodland species, 27 occurred at <10 sites overall (Table 3). The noisy miner was widespread and abundant, being recorded at 48 of the 52 sites (92%), including 24 cross sections and 24 linear sites.

### Model selection

The most parsimonious model (lowest AIC value) for each response group included the configuration (linear or cross section) and the biotic interaction (mean abundance of noisy miners) hypotheses (Table 2); while the woodland-dependent model also included the tree size hypothesis, and the species movement (birds flying along) model included the habitat amount hypothesis (Table 2). The deviance explained (R^2^) by these models was 53% for all woodland-dependent species, 36% for resident woodland species and 49% for species flying along transects (Table 2).

**Fig 3:**
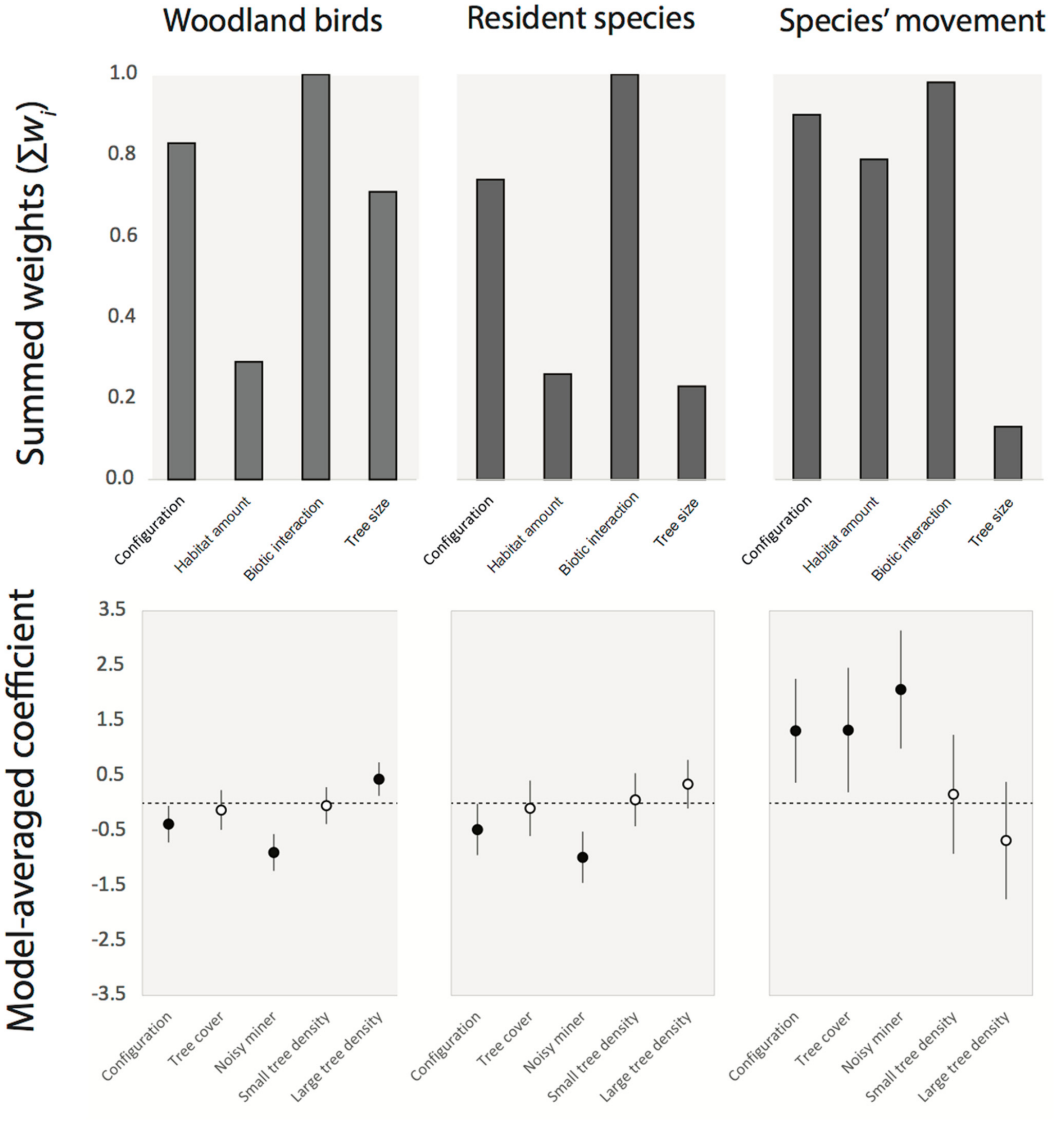
Summed Akaike weights and model-averaged coefficients (± standard error) of predictor groups for each of the response variables: a) *Woodland-dependent*, b) *Resident*, *c*) Species’ movement *Flying along*. Black circles indicate values for which the confidence intervals of coefficients do not overlap with zero. The configuration variable is tested in the model by using cross section as the reference category. Therefore, a negative coefficient for this variable implies that linear strips have fewer species, or in the case of species’ movement, that more species are moving along linear strips.

However, for all three response variables, multiple models had substantial support (i.e. Δ_i_ ≤2) (Table 2) and there was no ‘clearly best’ model (i.e. *w*_i_ > 0.90). Consequently, model averaging was performed to gain an understanding of the direction and size of the effect of each predictor variable in relation to each response variable. Summed Akaike weights (∑*w*_i_) and model-averaged coefficients for predictor variables are shown in Fig 3.

### Relative importance of hypotheses for response groups

#### Woodland-dependent species

**Fig 4:**
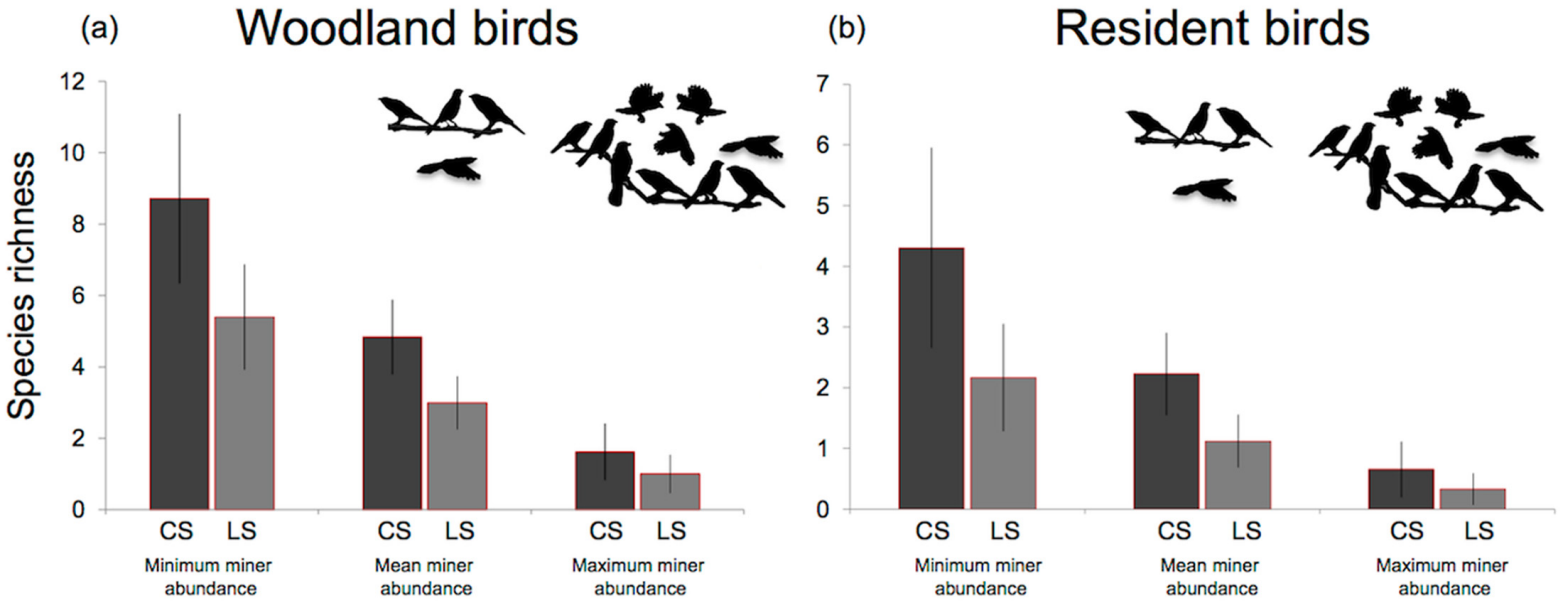
Predicted contribution of cross section (n = 26) and linear strips (n = 26) to species richness for response variables: a) *Woodland-dependent* (under different levels of noisy miner abundance), b) *Resident* (under different levels of noisy miner abundance). Bird silhouettes show numbers of noisy miners present under each category, averaged over four surveys (min: n = 0, mean: n = 4, max: n = 10).

The configuration hypothesis was well supported for woodland-dependent species (∑*w*_i_ = 0.83), with model averaging revealing that species richness was greater at cross sections than linear strips (i.e. a negative coefficient for linear strips, with cross section used as the reference category) (Figs 3 & 4). The tree size hypothesis was also well supported (∑*w*_i_ = 0.71), with parameter estimates revealing a positive association with the density of trees >30 cm diameter (Fig 3). Small trees (<30 cm) were less influential (Fig 3). The habitat amount hypothesis had little support (∑*w*_i_ = 0.29), with model-averaged coefficients overlapping zero.

#### Resident species

The configuration hypothesis was well supported for resident species (∑*w*_i_ = 0.74), with model averaging revealing that species richness was greater at cross sections than linear strips (i.e. a negative coefficient for linear strips, with cross section used as the reference category) (Figs 3 & 4). The tree size (∑*w*_i_ = 0.23) and habitat amount hypotheses received little support (∑*w*_i_ = 0.26). Model-averaged coefficients for these hypotheses overlapped with zero, indicating little influence on the residency of woodland birds at these sites.

#### Species movement

**Fig 5:**
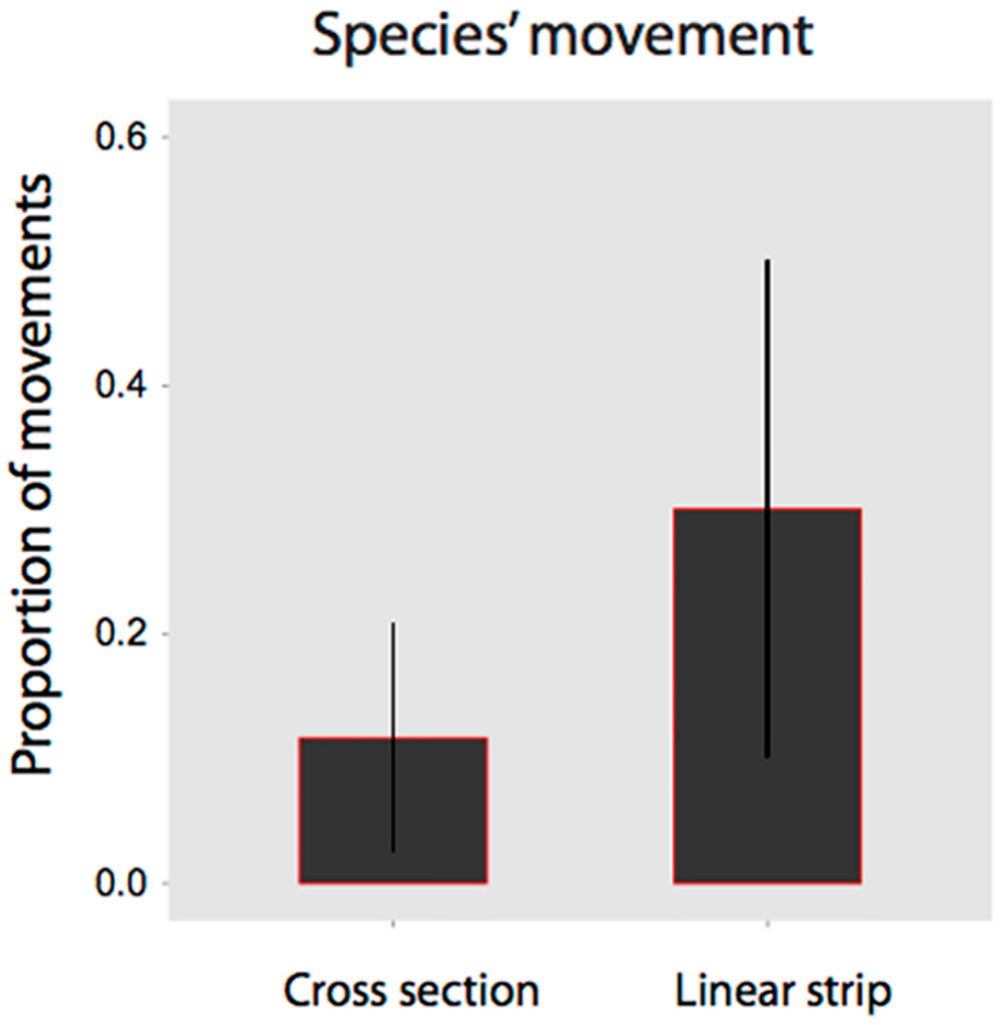
Predicted contribution of cross section (n = 26) and linear strips (n = 26) to species’ movement *Flying along* roadside vegetation.

The configuration hypothesis was well supported for woodland-dependent species flying along transects (∑*w*_i_ = 0.89). Parameter estimates reveal the proportion of woodland species at a site observed flying along roadside vegetation was greater for linear strips (Figs 3 & 5). The tree size hypothesis was not well supported for this group (∑*w*_i_ = 0.13), with coefficients overlapping zero for both small and larger trees (Fig 3). The habitat amount hypothesis was well supported (∑*w*_i_ = 0.79). Model-averaged coefficients revealed that species were responding to a greater level of tree cover within the landscape, particularly whilst flying along linear roadside strips (Fig 3).

#### Biotic interaction: the influence of the noisy miner

**Fig 6:**
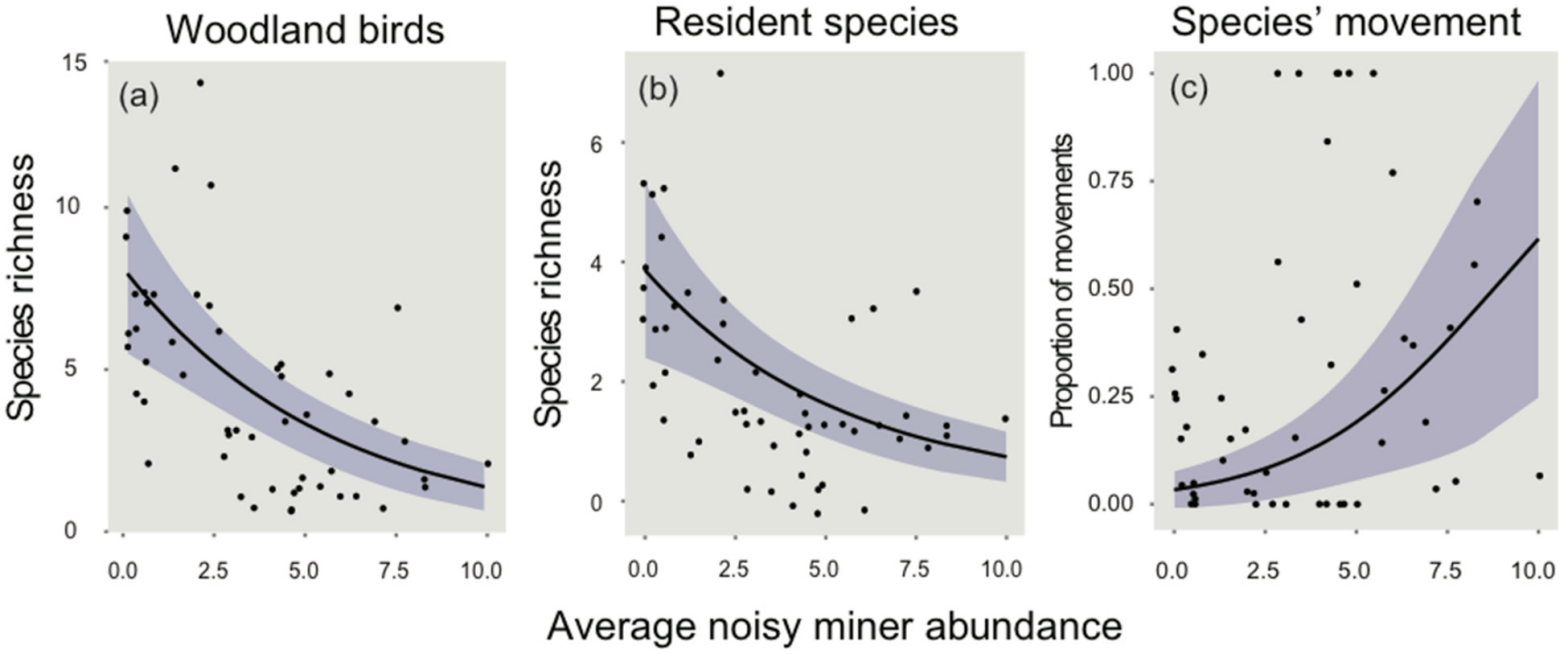
Predicted species richness of response groups of woodland birds (± standard error) in relation to mean abundance of the noisy miner: a) *Woodland-dependent*, b) *Resident*, c) Species movement *Flying along* the transect.

The biotic interaction hypothesis had a high level of support for all response groups: woodland-dependent (∑*w*_i_ = 1.0), resident (∑*w*_i_ = 1.0) and species movement (∑*w*_i_ = 0.98) (Fig 3). Model averaging revealed that noisy miner abundance had an important influence on all response groups (Fig 3). In each case, richness for response groups decreased as noisy miner abundance at sites increased; both when taking into consideration the treatment effect of configuration (Fig 4) and when pooling all sites regardless of treatment (Fig 6). For example, the richness of woodland-dependent species was predicted to decline from approximately eight species per site when no noisy miners were present, to just two with high abundance of noisy miners (Figs 4 & 6). In contrast, the proportion of woodland species observed flying along a transect increased by around 60% as noisy miner abundance increased from low to high (Fig 6).

## Discussion

Agricultural landscapes worldwide are characterised by linear networks of vegetation [7,19,42]. As these networks can comprise a large proportion of the remaining native vegetation [4,43], it is important to understand their role for biodiversity conservation. Here, we found that the configuration of roadside vegetation affects woodland bird species in a heavily modified agricultural region of southeastern Australia. Cross sections of roadside vegetation had more woodland bird species than did linear sections. The structure of roadside vegetation also influenced the biodiversity value, as the overall richness of woodland birds was positively associated with the density of larger trees. However, the potential value of roadside vegetation for woodland bird conservation is not currently being met due to a widespread aggressive species, the noisy miner, whose negative impact on all species’ groups exceeded that of all other predictor variables.

### Factors influencing woodland species in roadside vegetation

Our finding that intersections had greater numbers of woodland birds compared to linear sections is consistent with the ‘intersection effect’ reported by Lack [23]. The intersection effect predicts enhanced foraging efficiency by species at intersections compared with linear strips due to intersections having a greater number of movement pathways, both to vegetation surrounding the intersection and to other sources further along the network (Fig 1). This could facilitate easier access to food, particularly for species reluctant to use open spaces [24,27]. As this is the first study, of which we are aware, that has examined the intersection effect for roadside vegetation dominated by tree species (*cf*. shrub-dominated hedgerows and live-fences), we must infer similarities between our findings and those of studies conducted with different types of linear networks (e.g. [24,44]). Other factors may also come into play, such as the width, structure and composition of vegetation [4,7,45]; however, our sites were chosen to be similar with regard to these factors. These results strongly align with the intersection effect recorded for a variety of linear networks worldwide [23,27,46].

The conservation value of intersections was further affirmed by greater residency of woodland species in cross sections compared with linear strips, indicating that intersections are more than movement pathways, and may comprise important permanent habitat for woodland birds. We observed a greater proportion of individual woodland birds moving ‘along’ linear sections (*cf* cross sections). This finding further underscores the differing functional roles of intersections and linear strips, with the former having a higher potential to act as habitat and the latter being utilized to a greater extent as movement pathways [47].

The tree-size structure of roadside vegetation also affected woodland birds. A greater number of woodland species were found at sites with a higher density of larger trees (>30 cm diameter). Very large trees (typically those >60 cm diameter) in the study region often pre-date European settlement [48] and were relatively rare, comprising around 10 per cent of all trees recorded at sites. However, given their size, they are likely contributing disproportionally to the overall canopy cover within sites. Large eucalypt trees are considered ‘keystone structures’ in agricultural landscapes of southern Australia [49,50] as they provide resources for many biota (including woodland birds; [51]), such as tree hollows, perches and food [52–54]. The continued protection and provision of large trees in roadside vegetation is vital to woodland bird conservation in southern Australia.

An unexpected result of this study was that the amount of tree cover surrounding sites did not strongly influence the response groups, other than positively influencing the proportion of species observed flying along transects. Some species may have shown a preference for sites along linear strips with greater surrounding tree cover whilst flying, for the shelter, refuge or foraging resources the surrounding cover provides, or because these strips act as movement pathways between more highly connected permanent habitat patches. In this region, the extent of tree cover surrounding a site [29] and across the landscape [31] is an important driver of woodland bird richness. Here, the study sites were amongst highly modified farmland: the average tree cover in a 500 m buffer was 13%, and sites were on average 16.5 km from the closest relatively large (>40 ha) woodland remnant. In these relatively isolated sites, the connected nature of the roadside vegetation may be more important than low levels of surrounding tree cover for species richness of woodland species.

### The impact of the noisy miner

The aggressive native species, the noisy miner, consistently exerted the greatest influence on woodland bird species richness at sites. The impact of the noisy miner on woodland bird communities is well documented and supported by both correlative [55,56] and experimental [57] studies. The richness of all response groups declined as the mean abundance of noisy miners at a site increased. Noisy miners were common across the study region, being present at 48 of 52 sites, supporting previous findings of this species’ preference for edge habitats, such as roadside networks [58].

The dominance of noisy miners across these rural landscapes greatly diminishes the value of all linear elements (i.e. both cross section and linear strips) to woodland bird conservation. The number of woodland bird species recorded in linear elements with high abundance of noisy miners is just 19% of that at sites at which no noisy miners were recorded. Even stronger results were evident for richness of resident species: the predicted number of resident species occupying sites in roadside networks was 85% lower in sites with high abundance of noisy miners compared with unoccupied sites. There also was evidence that noisy miners altered movement patterns of woodland species. Species were more often seen to be moving ‘along’ linear elements (as opposed to perching, roosting or foraging at sites) when noisy miners were abundant. Together, these results suggest that where noisy miners are abundant the available habitat for woodland birds is greatly reduced, likely leading to further isolation of populations as they seek to find suitable habitat but avoid areas dominated by the noisy miner [33,59].

Previous studies have suggested that the negative impacts of the noisy miner can be ameliorated by two primary means: 1) by habitat restoration, specifically increasing the amount of understory vegetation, to which noisy miners respond negatively [60]; or 2) by direct removal of noisy miners (i.e. culling) [57]. Habitat restoration has the dual benefit of improving habitat quality for a range of taxonomic groups [61–63], while simultaneously minimising the impacts of noisy miners. However, it is also a longer-term solution, as restoration of understory vegetation can take years or decades [64,65]. Culling is a more immediate solution which could provide woodland bird communities with a reprieve, particularly given that such communities have recently been affected by a severe, long-term drought (the Millennium Drought, 2001–2009) [29]. Experimental removal of noisy miners [57] led to a rapid increase in the abundance and diversity of woodland birds. Management options that require ongoing and continuous intervention are not desirable as long-term solutions. Thus, a combination of understory restoration and culling over the short-term could provide woodland birds with opportunity to recolonise sites and persist in the longer-term.

### Enhancing the value of linear networks for fauna

Our findings have several clear implications for enhancing the conservation value of linear networks in modified agricultural regions. First, protection, maintenance and restoration of vegetation associated with the intersections of linear strips will have value by targeting these key locations in the linear network. This can be complemented by restoration of native vegetation in farm paddocks across the corners of intersections, thus creating larger ‘nodes’ of connected habitat. Second, the results support the benefits of protecting and retaining larger trees along roadsides to enhance the conservation value of roadside vegetation for woodland birds. Third, the restoration of a complex understory, combined with a program of large-scale removal of noisy miners, could reduce the detrimental effect of this species in the short term and substantially increase the effective area of habitat for woodland birds in rural landscapes.

## Supporting Information

S1 Table All birds recorded on transect over the four survey rounds, showing habitat association, foraging guild, presence and abundance at sites. ** OT = Open tolerant, OC = Open country, Wdl = Woodland-dependent. + I = Insectivore, P = Predatory, N = Nectarivore, S = Granivore, F = Frugivore, V = Vegetation, R = Raptorial.
(DOCX)

S1 Data (XLSX)
