# Supplementary material for: At the Crossroads: Does the Configuration of Roadside Vegetation Affect Woodland Bird Communities in Rural Landscapes?
Source: PLoS One. 2016 May 16;11(5):e0155219. doi: 10.1371/journal.pone.0155219 (PMC4868280; doi:10.1371/journal.pone.0155219)
Supplement: S1 Table — ** OT = Open tolerant, OC = Open country, Wdl = Woodland-dependent. + I = Insectivore, P = Predatory, N = Nectarivore, S = Granivore, F = Frugivore, V = Vegetation, R = Raptorial. (DOCX) [file pone.0155219.s001.docx]

# Supporting Information

**S1 Table: All birds recorded on transect over the four survey rounds, showing habitat association, foraging guild, presence and abundance at sites.**

| *Common Name* | *Scientific Name* | *Habitat*** | *Foraging*  *Guild+* | *Total number of sites (n=52)* | *% Sites* | *Total*  *Observations* |
| --- | --- | --- | --- | --- | --- | --- |
|  |  |  |  |  |  |  |
| Australian king parrot | *Alisterus scapularis* | OT | S | 1 | 2 | 3 |
| Australian magpie | *Gymnorhina tibicen* | OC | I | 50 | 96 | 306 |
| Australian raven | *Corvus coronoides* | OC | P | 18 | 35 | 44 |
| Black-chinned honeyeater | *Melithreptus gularis* | Wdl | N | 1 | 2 | 3 |
| Black-faced cuckoo-shrike | *Coracina novaehollandiae* | OT | I | 2 | 4 | 2 |
| Black-shouldered kite | *Elanus axillaris* | OC | R | 3 | 6 | 4 |
| Blue-faced honeyeater | *Entomyzon cyanotis* | Wdl | N | 1 | 2 | 1 |
| Brown falcon | *Falco berigora* | OC | R | 2 | 4 | 2 |
| Brown quail | *Coturnix australis* | Wdl | S | 1 | 2 | 5 |
| Brown treecreeper | *Climacteris picumnus* | Wdl | I | 2 | 4 | 21 |
| Brown-headed honeyeater | *Melithreptus brevirostris* | Wdl | N | 4 | 8 | 48 |
| Common bronzewing | *Phaps chalcoptera* | Wdl | S | 3 | 6 | 18 |
| Crested pigeon | *Ocyphaps lophotes* | OC | S | 14 | 27 | 37 |
| Crested shrike-tit | *Falcunculus frontatus* | Wdl | I | 3 | 6 | 7 |
| Crimson rosella | *Platycercus elegans* | Wdl | S | 2 | 4 | 3 |
| Dusky woodswallow | *Artamus cyanopterus* | Wdl | I | 1 | 2 | 8 |
| Eastern rosella | *Platycercus eximius* | OT | S | 49 | 94 | 737 |
| Eastern spinebill | *Acanthorhynchus tenuirostris* | Wdl | N | 1 | 2 | 1 |
| European goldfinch* | *Carduelis carduelis* | OC | S | 1 | 2 | 1 |
| Fairy martin | *Hirundo ariel* | OC | I | 1 | 2 | 6 |
| Flame robin | *Petroica phoenicea* | OT | I | 12 | 23 | 116 |
| Galah | *Cacatua roseicapilla* | OC | S | 48 | 92 | 695 |
| Golden whistler | *Pachycephala pectoralis* | Wdl | I | 10 | 19 | 18 |
| Grey butcherbird | *Cracticus torquatus* | OT | P | 8 | 15 | 13 |
| Grey currawong | *Strepera versicolor* | OT | P | 1 | 2 | 1 |
| Grey fantail | *Rhipidura fuliginosa* | Wdl | I | 18 | 35 | 34 |
| Grey shrike-thrush | *Colluricincla harmonica* | Wdl | I | 12 | 23 | 20 |
| Grey-crowned babbler | *Pomatostomus temporalis* | Wdl | I | 7 | 13 | 56 |
| Jacky winter | *Microeca fascinans* | Wdl | I | 2 | 4 | 6 |
| Laughing kookaburra | *Dacelo novaeguineae* | OT | P | 13 | 25 | 24 |
| Little raven | *Corvus mellori* | OC | P | 16 | 31 | 42 |
| Long-billed corella | *Cacatua tenuirostris* | OC | S | 4 | 8 | 31 |
| Magpie-lark | *Grallina cyanoleuca* | OC | I | 7 | 13 | 14 |
| Mistletoebird | *Dicaeum hirundinaceum* | Wdl | F | 1 | 2 | 2 |
| Musk lorikeet | *Glossopsitta concinna* | Wdl | N | 31 | 60 | 728 |
| Nankeen kestrel | *Falco cenchroides* | OC | R | 2 | 4 | 4 |
| Noisy friarbird | *Philemon corniculatus* | Wdl | N | 1 | 2 | 8 |
| Noisy miner | *Manorina melanocephala* | OT | N | 48 | 92 | 728 |
| Olive-backed oriole | *Oriolus sagittatus* | Wdl | I | 1 | 2 | 1 |
| Pied butcherbird | *Cracticus nigrogularis* | OT | P | 1 | 2 | 1 |
| Pied currawong | *Strepera graculina* | Wdl | P | 7 | 13 | 11 |
| Red wattlebird | *Anthochaera carunculata* | Wdl | N | 12 | 23 | 125 |
| Red-rumped parrot | *Psephotus haematonotus* | OT | S | 17 | 33 | 117 |
| Restless flycatcher | *Myiagra inquieta* | OT | I | 9 | 17 | 21 |
| Rufous whistler | *Pachycephala rufiventris* | Wdl | I | 5 | 10 | 7 |
| Scarlet robin | *Petroica multicolor* | Wdl | I | 4 | 8 | 7 |
| Speckled warbler | *Chthonicola sagittata* | Wdl | I | 1 | 2 | 1 |
| Spotted pardalote | *Pardalotus punctatus* | Wdl | I | 2 | 4 | 3 |
| Striated pardalote | *Pardalotus striatus* | OT | I | 40 | 77 | 263 |
| Sulphur-crested cockatoo | *Cacatua galerita* | OT | S | 11 | 21 | 80 |
| Superb fairy-wren | *Malurus cyaneus* | Wdl | I | 5 | 10 | 49 |
| Tree martin | *Hirundo nigricans* | Wdl | I | 7 | 13 | 42 |
| Varied sittella | *Daphoenositta chrysoptera* | Wdl | I | 6 | 12 | 41 |
| Wedge-tailed eagle | *Aquila audax* | OC | R | 3 | 6 | 3 |
| Welcome swallow | *Hirundo neoxena* | OC | I | 7 | 13 | 32 |
| White-bellied cuckoo-shrike | *Coracina papuensis* | Wdl | I | 1 | 2 | 1 |
| White-browed babbler | *Pomatostomus superciliosus* | Wdl | I | 1 | 2 | 5 |
| White-plumed honeyeater | *Lichenostomus penicillatus* | Wdl | N | 31 | 60 | 297 |
| White-winged chough | *Corcorax melanorhamphos* | Wdl | I | 19 | 37 | 282 |
| Willie wagtail | *Rhipidura leucophrys* | OT | I | 20 | 38 | 70 |
| Yellow thornbill | *Acanthiza nana* | Wdl | I | 12 | 23 | 120 |
| Yellow-faced honeyeater | *Lichenostomus chrysops* | Wdl | N | 1 | 2 | 1 |
| Yellow-plumed honeyeater | *Lichenostomus ornatus* | Wdl | N | 1 | 2 | 1 |
| Yellow-rumped thornbill | *Acanthiza chrysorrhoa* | OT | I | 10 | 19 | 144 |

** OT = Open tolerant, OC = Open country, Wdl = Woodland-dependent

+ I = Insectivore, P = Predatory, N = Nectarivore, S = Granivore, F = Frugivore, V = Vegetation, R = Raptorial
